# Single-molecule Force Spectroscopy Predicts a Misfolded, Domain-swapped Conformation in human γD-Crystallin Protein*

**DOI:** 10.1074/jbc.M115.673871

**Published:** 2015-12-24

**Authors:** Sergi Garcia-Manyes, David Giganti, Carmen L. Badilla, Ainhoa Lezamiz, Judit Perales-Calvo, Amy E. M. Beedle, Julio M. Fernández

**Affiliations:** From the ‡ Department of Physics and Randall Division of Cell and Molecular Biophysics, King's College London, London WC2R 2LS, United Kingdom and; the § Department of Biological Sciences, Columbia University, New York, New York 10027

**Keywords:** atomic force microscopy (AFM), cataract, crystallin, protein misfolding, single-molecule biophysics

## Abstract

Cataract is a protein misfolding disease where the size of the aggregate is directly related to the severity of the disorder. However, the molecular mechanisms that trigger the onset of aggregation remain unknown. Here we use a combination of protein engineering techniques and single-molecule force spectroscopy using atomic force microscopy to study the individual unfolding pathways of the human γD-crystallin, a multidomain protein that must remain correctly folded during the entire lifetime to guarantee lens transparency. When stretching individual polyproteins containing two neighboring HγD-crystallin monomers, we captured an anomalous misfolded conformation in which the β1 and β2 strands of the N terminus domain of two adjacent monomers swap. This experimentally elusive domain-swapped conformation is likely to be responsible for the increase in molecular aggregation that we measure *in vitro*. Our results demonstrate the power of force spectroscopy at capturing rare misfolded conformations with potential implications for the understanding of the molecular onset of protein aggregation.

## Introduction

Protein misfolding and aggregation have been identified as critical biochemical processes underlying the pathology of a variety of increasingly prevalent human diseases, including amyotrophic lateral sclerosis (1) and antitrypsin deficiency (2). For a number of these conditions, domain swapping has been proposed as the mechanism of the off-pathway aggregation reaction (3, 4). Lens cataract is one of the most conspicuous protein misfolding diseases, affecting nearly 50% of the population over 75 years of age, and is the leading cause of blindness in the world (5). Crystallins, which are synthesized *in utero*, comprise 90% of proteins in the mature lens and guarantee its transparency (6–9). These singular physiological conditions impose severe stability constraints on the crystallin proteins, whose functionality relies on the retention of a life-long soluble, folded state (10).

**FIGURE 1.**
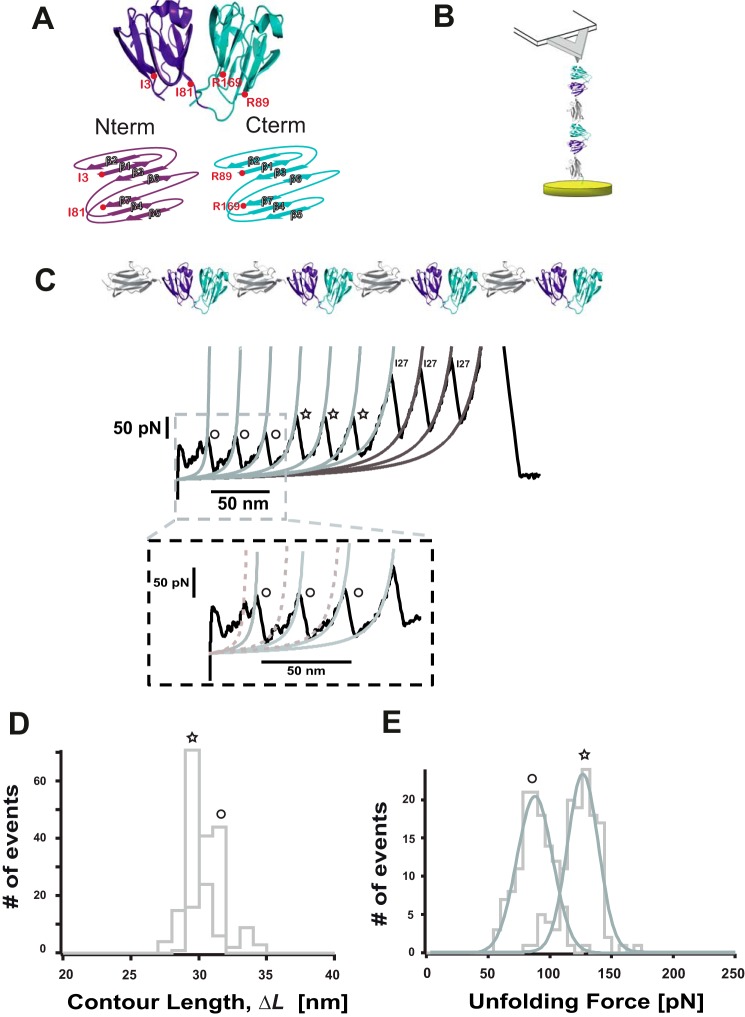
**The two domains of HγD-crys unfold independently following a hierarchy in their mechanical stability.** *A*, structure of a HγD-crys monomer. *Nterm*, N terminus; *Cterm*, C terminus. *B*, single-molecule AFM experiment layout. *C*, the unfolding trajectory of an individual (HγD-crys-I27)_4_ polyprotein follows a hierarchy in the mechanical stability whereby the low mechanical stability events (*circles*) unfold first (*D*) at 84.7 ± 14.50 pN (*n* = 123), followed by those exhibiting higher mechanical resistance (*stars*) at 123.6 ± 12.55 pN (*n* = 127). *E*, by contrast, both types of events cannot be singled out on the basis of their increment in contour length, which is in both cases Δ*L_c_* ∼30 nm (Δ*L_c_* = 29.1 ± 0.62 nm, *n* = 120 (*stars*), and Δ*L_c_* = 30.4 ± 0.8 nm, *n* = 116 (*circles*)).

Cataractous lenses extracted surgically from the human eye contain insoluble species that include the three main groups of crystallins found in vertebrates (9), including the α-, β-, and γ-crystallin families (11). Although α-crystallin exhibits chaperone activity, β- and γ-crystallins have a largely structural function (7), providing the lens with the high refractive index required for clear vision (6, 12). Human γ D-crystallin (HγD-crys)^2^ is the third most abundantly expressed γ-crystallin in the nucleus of the adult lens and accumulates in mature-onset cataracts. The x-ray crystal structure (Fig. 1*A*, PDB code 1HK0) (13) reveals that this 173-residue protein consists of a C-terminal domain (Ctd, *blue*) and N-terminal domain (Ntd, *purple*) joined by a seven-amino acid linker hinge. Both are composed of two β-sheet Greek key motifs, and they share a highly conserved hydrophobic interface (14). Biochemical studies have shown that the two domains exhibit different thermodynamic stability, with the Ctd being more stable than the Ntd. Parallel unfolding/refolding experiments of WT HγD-crys identified a partially folded intermediate in which the Ctd is native-like, but the Ntd is not fully folded (15, 16). Such a partially folded conformation initiated an irreversible aggregation pathway that competed with productive refolding under physiological conditions. The HγD-crys aggregates exhibited an ordered nature with a filamentous appearance (15). By contrast, when exposed to low pH, the resulting aggregates showed amyloid characteristics (17). In this vein, amyloid fibrils were also observed in recombinant mutant γB-crystallin under physiological buffer conditions (18).

Although these biochemical assays have provided interesting morphological insights into the high molecular weight insoluble species (15, 17), the molecular basis governing the *in vitro* onset of HγD-crys polymerization (19) remains poorly understood (10). In this respect, recent molecular dynamics simulations have indeed postulated that HγD-crys may polymerize through successive domain swapping of the C-terminal β strands (20). However, direct experimental determination of such dimeric conformation remains elusive.

Here we use a combination of molecular biology engineering techniques and single-molecule force spectroscopy AFM to characterize the unfolding mechanism of the multidomain HγD-crys protein. Single-molecule mechanical experiments have mapped the force-induced unfolding pathways of a wide variety of proteins along a well defined end-to-end reaction coordinate (21, 22). Crucially, these experiments have been able to identify misfolding events along the individual unfolding trajectories of topologically simple small proteins (23–27). By engineering HγD-crys polyprotein constructs in which the number of neighboring HγD-crys domains is changed rationally, we unambiguously identify a dimeric conformation in which two N termini β strands swapped. We hypothesize that such an elusive conformation might hold the key to explaining, from a single molecule perspective, the genesis of mature-onset cataracts, a widespread misfolding disease.

## Experimental Procedures

### 

#### 

##### Protein Engineering

The polyproteins used in this work (HγD-crys-I27)_4_, (I27-Ntd)_4_, (I27-Ctd)_4_, [I27-(HγD-crys)_2_-I27], (HD-crys_R14C_-I27)_4_, [I27-(HγD-crys_R14C_)_2_-I27], and (HγD-crys-I27)_2_ were subcloned using the BamHI, BglII, and KpnI restriction sites (28). The multidomain proteins were cloned into the pQE80L (Qiagen) expression vector and transformed into the BLRDE3 *Escherichia coli* expression strain. Cells were grown in Luria broth supplemented with 100 μg/ml ampicillin at 37 °C. After reaching an *A*_600_ of ∼0.6, cultures were induced with 1 mm isopropyl 1-thio-β-d-galactopyranoside and incubated overnight at 25 °C. Cells were disrupted with a French press, and the polyproteins from the lysate were purified by metal affinity chromatography on Talon resin (Takara, Clontech), followed by gel filtration using a Superdex 200 10/300 GL column (GE Biosciences). Proteins were stored in PBS buffer at 4 °C. The [I27-(HγD-crys)_2_-I27] polyprotein with a longer linker was constructed by adding the sequence GGCTCTGGTTCAGGTTCAGGAAGCGGCAGTGGTTCAGGAAGC to the linker connecting both HγD-crys monomers.

##### Force Spectroscopy

Constant velocity atomic force microscopy experiments were conducted at room temperature using both a home-made setup described elsewhere (29) and a commercial Luigs and Neumann force spectrometer (30). In all cases, the sample was prepared by depositing 1–10 μl of protein in PBS solution (at a concentration of 1–10 mg ml^−1^) onto a freshly evaporated gold coverslide. Each cantilever (Si_3_N_4_ Bruker MLCT-AUHW) was calibrated individually using the equipartition theorem, giving rise to a typical spring constant of ∼12 pN nm^−1^. Single proteins were picked up from the surface and pulled at a constant velocity of 400 nm s^−1^. Experiments were carried out in phosphate-buffered saline solution (pH 7.2).

##### Data Analysis

All data were recorded and analyzed using custom software written in Igor Pro 6.0 (Wavemetrics). For the experiments conducted with the [I27-(HγD-crys)_2_-I27], [I27-(HγD-crys_R14C_)_2_-I27], and [I27-(HγD-crys)]_2_ proteins, only recordings showing the signature corresponding to the unfolding of both I27 modules were analyzed.

##### Light Scattering

Thermal unfolding of 0.6 mg/ml of the corresponding polyproteins was induced by incubation at 60 °C in PBS buffer. Aggregation of each individual polyprotein was monitored by continuous measurement of light scattering at 350 nm in a Fluoromax 4 (Horiba) instrument.

## Results

### 

#### 

##### Nanomechanical Characterization of the Multidomain HγD-crys Protein

**FIGURE 2.**
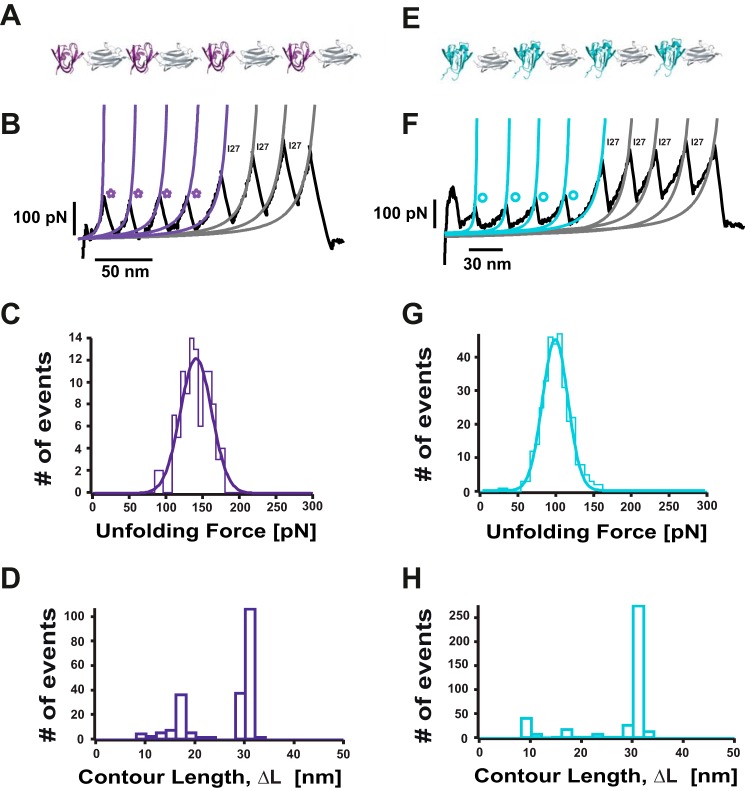
**The N terminus domain of HγD-crys exhibits a higher mechanical stability than the C terminus domain.** *A*, schematic of the (I27-Ntd)_4_ polyprotein. *B*, typical force extension unfolding trajectory of a (I27-Ntd)_4_ polyprotein, showing four unfolding peaks (*purple stars*) followed by the unfolding of the well characterized I27 protein (*gray fits*). *C*, distribution of unfolding forces for the Ntd, yielding an average unfolding force of 136.2 ± 18.0 pN (*n* = 106). *D*, distribution of the measured increment in contour length, Δ*L_c_*, upon unfolding (*n* = 217). Although a main unfolding Δ*L_c_* of 29.3 ± 0.7 nm is measured, other shorter Δ*L* values are also evident, probably because of unfolding events of partially unfolded domains. *E*, schematic of the (I27-Ctd)_4_ polyprotein. *F*, individual unfolding trajectory of an individual (I27-Ctd)_4_ polyprotein_,_ showing the unfolding of the Ctd first (*blue circles* and *blue* Worm-like chain fits), followed by the unfolding of the I27 protein (*gray* Worm-like chain fits). *G*, mechanical unfolding of an individual Ctd requires a force of 96.1 ± 16.8 pN (*n* = 331). *H*, the distribution of associated increment in contour length Δ*L_c_* values (*n* = 430) shows a main unfolding Δ*L_c_* = 29.9 ± 0.8 nm together with and a much smaller population of events with associated shorter Δ*L_c_* values.

A single polyprotein composed of four identical repeats of the HγD-crys monomer, each of them flanked by the well characterized I27th module of the titin protein (HγD-crys-I27)_4_, was tethered between the AFM cantilever tip and the gold substrate (Fig. 1*B*) and stretched at a constant velocity. The resulting unfolding trajectories (Fig. 1*C*) displayed a sawtooth pattern consisting of up to eight unfolding force peaks separated by an increment in contour length of Δ*L*_c_ ∼30 nm (Fig. 1*D*) corresponding to the unfolding of the HγD-crys that precedes the unfolding of the I27 molecular fingerprint (31). Crucially, the unfolding events clustered into two different levels of mechanical stability, of ∼85 pN (*circles*) and ∼125 pN (*stars*), as shown in Fig. 1*E*. The forces required to unfold the wild-type γD-crystallin polyprotein are similar to the forces required to unfold the Ca^2+^-binding archaeal M-crystallin (∼90 pN) (31). Interestingly, the low-force unfolding domains often exhibit a weak (∼50 pN) mechanical intermediate with a related increment in contour length of Δ*L_c_* ∼8 nm (Fig. 1*C*, *inset*). Because of the two-domain structure of the HγD-crys monomer (Fig. 1*A*), it is very likely that the two distinct levels of mechanical stability observed in Fig. 1 correspond to the independent, sequential unfolding of both domains (Ntd and Ctd). To unambiguously identify the origin of the low and high mechanical stability events, we individually tested the mechanical stability of the (I27-Ntd)_4_ and (I27-Ctd)_4_ polyproteins (Fig. 2, *A* and *E*, respectively). These experiments revealed that the low mechanical stability events (*blue circles*, Fig. 2*B*) correspond to the unfolding of the Ctd (∼96 pN, Fig. 2*G*), whereas the Ntd (*purple stars*) is more mechanically resilient (Fig. 2*B*), requiring ∼136 pN (Fig. 2*C*) to unfold. Together, these experiments demonstrate that, at the pulling velocity of 400 nm s^−1^, the mechanical unfolding of the HγD-crys monomer occurs sequentially, where the Ctd unfolds first, followed by the unfolding of the more mechanically resilient Ntd. The close agreement between the measured unfolding forces for the complete HγD-crys monomer and for each of the truncated forms suggests that each domain remains stable on its own and unfolds independently after the disruption of the binding hydrophobic interface. This observation is surprising because it contrasts with chemical denaturation experiments demonstrating that the hydrophobic interface determines the relative stability of the C and N termini in βB2- and γB-crystallin (32, 33). To further prove the putative mechanical role of the hydrophobic interface, we studied the mechanical stability of the polyprotein mutant containing the double mutation Gln-54/Gln-143, which has been described to chemically destabilize the hydrophobic interface (34). Our experiments demonstrate that the mechanical stability of the mutant protein does not differ from that of the wild-type protein (data not shown). The unfolding sequence observed in our nanomechanical experiments agrees with the predictions obtained by coarse grain simulations under force (35) and is in sharp contrast with the relative thermodynamic stability of both domains, showing that the Ctd is more stable than the Ntd (15).

##### HγD-crys Forms a Misfolded, Domain-swapped Conformation

**FIGURE 3.**
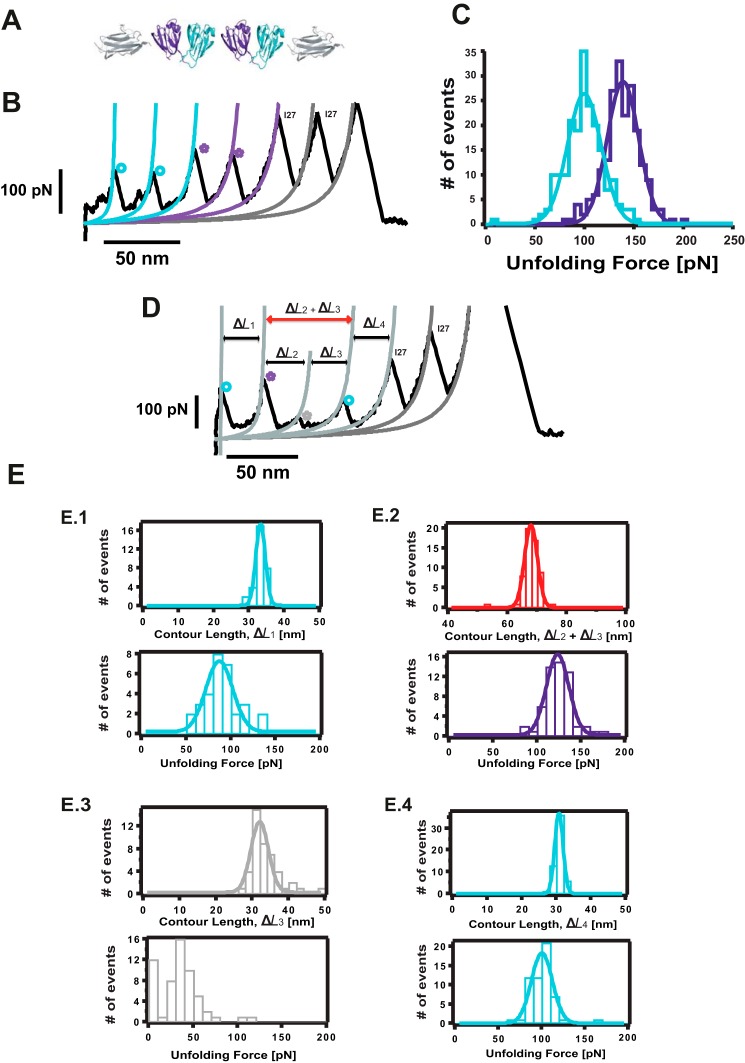
**Identification of a misfolded dimeric conformation in HγD-crys.** *A*, schematic of the constructed [I27-(HγD-crys)_2_-I27] polyprotein. Mechanical unfolding of this construct results in two main types of trajectories. *B*, in ∼85% of instances, the Ctd (*blue fit*) unfolds first at 96.7 ± 17.0 pN (*n* = 198), followed by the unfolding of the Ntd (*purple fit*), at 135.8 ± 16.0 pN (*n* = 201) before the unfolding of the I27 marker occurring at 251.9 ± 34.0 pN (*n* = 158). *D*, in the remaining ∼15% of trajectories (*n* = 57), the [I27-(HγD-crys)_2_-I27] polyprotein unfolds in an anomalous fashion that is not consistent with a mechanical hierarchy scenario. *E*, a first unfolding peak (*E.1*) occurs at 82.2 ± 15.0 pN (*n* = 31) and Δ*L*_1_ = 32.3 ± 1.3 nm (*n* = 31). The second unfolding peak (*purple star*) occurs at 118.4 ± 12.8 pN and (Δ*L*_2_ + Δ*L*_3_) = 66.9 ± 2.1 nm; *n* = 57, *E.2*). Next, a low mechanical stability peak of ∼35 pN and Δ*L*_3_ = 31.0 ± 2.4 nm (*n* = 45) is observed. Prior to the unfolding of the I27 internal controls, a last unfolding event, characterized by an unfolding force of 95.3 ± 12.1 pN and a Δ*L*_4_ = 29.7 ± 1.2 nm (*n* = 56), is observed (*E.4*).

Protein aggregation requires, by definition, the physical interaction of individual monomers present in solution. To increase the effective monomer concentration in the context of a single polyprotein chain, we engineered a construct consisting of two neighboring (HγD-crys) domains flanked by one I27 monomer at each side (Fig. 3*A*). Pulling on the resulting [I27-(HγD-crys)_2_-I27] polyprotein results in two types of unfolding trajectories. In the majority of cases (∼85%), a “regular” unfolding is observed (Fig. 3*B*), following the same pattern as the (HγD-crys-I27)_4_ polyprotein, where the forces to unfold both the Ctd (∼97 pN, Fig. 3*B*, *blue fits*, *circles*) and Ntd (∼137 pN, Fig. 3*B*, *purple fits*, *stars*, and Fig. 3*C*) closely match the forces measured for each domain independently (Fig. 2, *C* and *G*). In addition to the expected 30-nm increment in contour length typically observed for both domains unfolding, in some instances, HγD-crys unfolds eliciting shorter lengths, revealing the presence of low-probability alternative unfolding pathways (data not shown).

Surprisingly, in the remaining ∼15% of cases, the [I27-(HγD-crys)_2_-I27] polyprotein unfolds following an “anomalous” pattern (Fig. 3*D*). The first HγD-crys unfolding event is characterized by an unfolding force of 82 pN and an associated increment in contour length of Δ*L*_1_ ∼30 nm (Fig. 3, *E.1*), reminiscent of the unfolding of a Ctd (Fig. 3*D, blue circle*). This is followed by an unfolding event occurring at higher forces (∼120 pN) (Fig. 3, *E.2*), close to the force value that is required to unfold an Ntd (Fig. 3*D, purple star*) and associated with an increment in contour length Δ*L*_2_. Notably, a clear unfolding peak of low mechanical stability (∼35 pN) occurs next, releasing a protein length Δ*L*_3_ ∼31 nm (Fig. 3, *E.3*). The last HγD-crys unfolding event occurs at a force of ∼ 95 pN, again compatible with the unfolding of the second Ctd (Fig. 3*D, blue circle*, Δ*L*_4_ ∼30 nm) (Fig. 3, *E.4*). This unfolding pattern is evocative of a mechanism whereby at least one mechanically labile domain remains protected from the effect of force until a more resilient domain unfolds first (36). In 12 of the 57 anomalous trajectories, the force corresponding to the unexpectedly low-stability peak is too low to be measured, in which case a large associated increment in contour length of (Δ*L*_2_ + Δ*L*_3_) ∼67 nm is measured after the unfolding of the Ntd. For the rest of the trajectories, application of the extended Kalman filter algorithm (37) provided rigorous, real-time estimates of Δ*L*_3._

**FIGURE 4.**
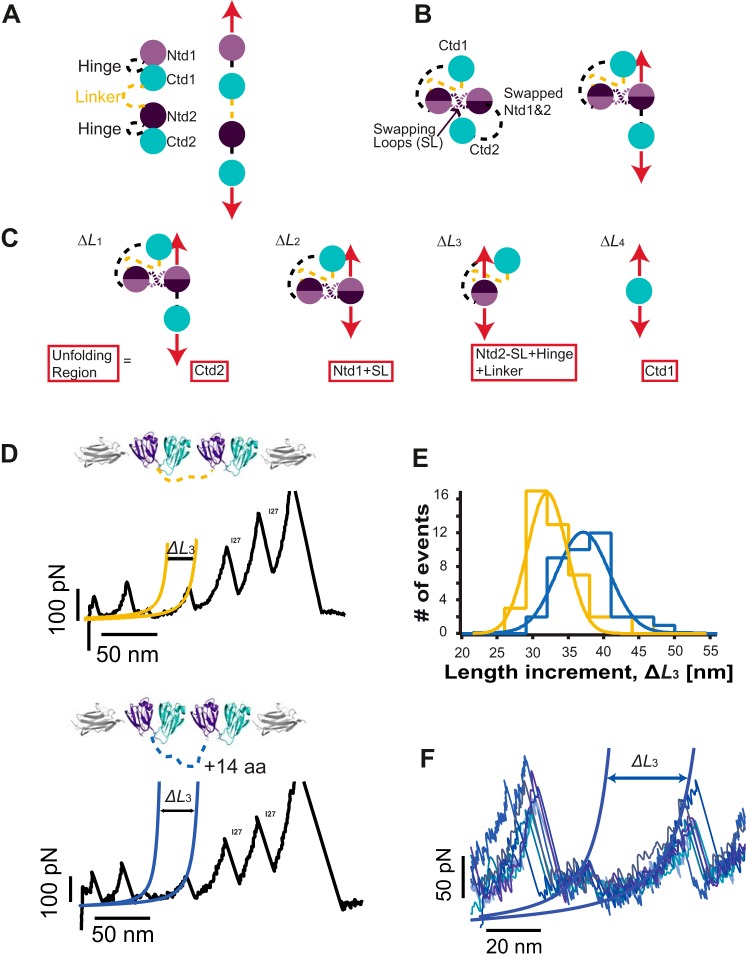
**A simple structural model compatible with stretching a domain-swapped conformation.** *A*, schematic explaining the regular unfolding trajectories. *B*, schematic of the polyprotein conformation compatible with the anomalous trajectories on the basis of Ntd domain swapping. When exposed to mechanical force (*red arrows*), this “swapped” conformation acts as a force-shielding element that prevents Ctd1 from being exposed to the mechanical force. *SL*, swapping loop. *C*, the force is first applied to the termini of Ctd2 and Ntd1. The unfolded and extended region is described in the *red frame* in each step along the sequence. *D*, pulling on the [I27-(HγD-crys)_2_-I27] polyprotein in which 14 extra residues, (GS)_7_, were added to the linker region (*dashed yellow lines*) displays swapped unfolding trajectories akin to those observed for the regular construct. *E*, comparing the increment in the protein contour length measured concomitant to the unfolding of the Ntd2 domain (Δ*L*_3_) for the regular polyprotein construct (*yellow fit*, Δ*L*_3_ = 30.5 ± 2.8 nm, *n* = 45) and for the longer linker polyprotein (*blue fit*, Δ*L*_3_ = 35.6 ± 3.7 nm, *n* = 38) reveals a measured ∼5.1-nm difference in length. *F*, pulling the longer linker polyprotein at a higher speed (1200 nm s^−1^) provides a clearer and more accurate measurement of Δ*L*_3_.

To explain the origin of the observed unfolding trajectories, we constructed a plausible schematic (Fig. 4). In the regular pattern (Fig. 4*A*), upon stretching the [I27-(HγD-crys)_2_-I27] polyprotein, all domains are immediately exposed to the pulling force. The unfolding sequence involves a first fast extension of both hinges connecting the Ntd and Ctd in each HγD-crys monomer together with the linker connecting both neighboring HγD-crys monomers. This is followed by the unfolding of both the Ctd and Ntd according to their relative mechanical stabilities.

Inspired by recent molecular dynamics simulations (20) and on the basis of the domain-swapped crystal structure of βB2-crystallin (38, 39), we first considered domain swapping as a putative structural motif that enables us to rationalize the origin of these anomalous unfolding trajectories that do not follow the classical sequential mechanical hierarchy scenario (36, 40). The observed trajectories are fully compatible with a simple structural model where the swap occurs between the Ntds (Fig. 4*B*) and could not be explained when the swap occurred in the Ctd instead. According to this model, when stretching the swapped dimer, the mechanical force is first applied to Ntd1 and Ctd2 (Fig. 4*C*). Because of their different mechanical stabilities, Ctd2 has the highest probability to unfold first (Fig. 4*C*, Δ*L_1_*). The force is then applied between the N and C termini of the Ntd1 swapped structure (Fig. 4*C*, Δ*L_2_*), which will trigger the whole Ntd1 to unfold and extend, together with an extra swapped linker (Fig. 4*C*, *dashed purple lines*). When unfolded, the force is applied directly to the remaining Ntd2 part of the swapped conformation. Unfolding and extending such an Ntd2 part of the swapped structure (Fig. 4*C*, Δ*L_3_*) occurs concomitant to the extension of the hinge linker that connects the Ctd and Ntd in the structure (Fig. 4*C*, *black dashed lines*), and also the linker (Fig. 4*C*, *yellow dashed lines*) connecting both HγD-crys monomers within the polyprotein chain. Finally, the remaining Ctd1 can unfold easily (Fig. 4*C*, Δ*L_4_*). Notably, in this polyprotein chimera, the interdomain linker is the only engineered (and, therefore, non-native) part of the construct. Investigating the effects of changing the linker length on these misfolding trajectories would enable us to unambiguously pinpoint the exact position in the unfolding sequence where the linker extension takes place (Fig. 4*B*, *yellow dashed lines*), further testing our domain swap hypothesis.

To this end, we constructed an analogous polyprotein, [I27-(HγD-crys)_2_-I27], where 14 extra amino acids were added to the linker between both individual HγD-crys domains. Pulling on such a construct also showed domain-swapped trajectories (Fig. 4*D*) exhibiting an unfolding pattern akin to that observed for the [I27-(HγD-crys)_2_-I27] polyprotein with the regular (shorter) linker. Indeed, comparing Δ*L*_3_ for both polyprotein constructs revealed that the unraveling of the Ntd2 swapped domain was ∼5.1 nm longer in the modified construct (Fig. 4*E*, Δ*L*_3_ ∼35.6 nm, *blue fit*), closely matching the expected length (14 amino acids × 0.4 nm/amino acid = 5.6 nm).

**FIGURE 5.**
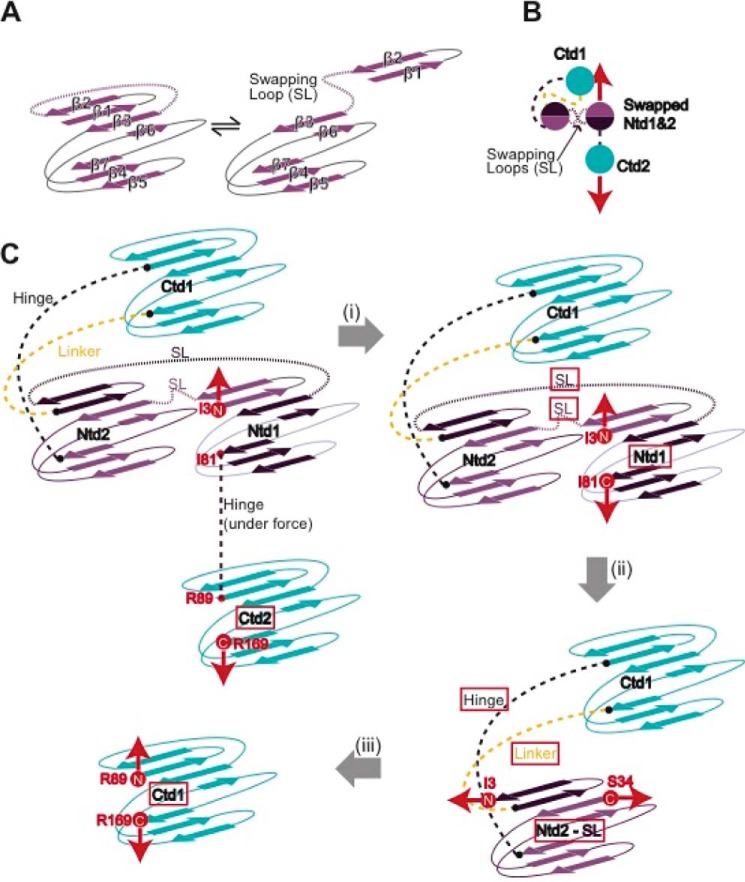
**The most probable swapping motif involves the β1 and β2 strands of the Ntd of HγD-crys.** *A*, topological model of the domain-swapped protein construct that identifies the swapping motifs between two Ntds (*B*) and highlights the sequence of structural rearrangements the polyprotein construct undergoes when exposed to mechanical force (*C*). After Ctd2 unfolding (*i*), the force is applied from the C-terminal of Ntd1 (*I81*) and the N terminus of the swapped conformation (*I3*). It is very possible that the swapping rearrangement accounts for the lower mechanostability (∼20 pN) of this swapped Ntd1 domain with respect to a WT Ntd (Fig. 2). When the Ntd1-swapped motif unfolds and extends concomitant to the swapping loop, the force is directly applied to the new N(I3) and C(S34) termini of the Ntd2 swapped conformation (*ii*). Remarkably, the pulling direction has now changed, which readily explains the lower mechanical stability of Ntd2 of the swapped structure. This plausible structural model is consistent with the experimental traces we observed (Fig. 3*D*) in terms of the unfolding sequence, increment in contour length, and measured unfolding force.

To precisely locate the position of the domain swap, we first took into account the following two structural considerations: in general, swapping motifs occur close to domain termini (41), and unstructured flexible regions lacking secondary structure enhance the swapping probability (4). We combined them with our experimental observations. As shown in Fig. 4*C*, the measured length Δ*L*_2_ corresponds to the unfolding and extension of Ntd1 together with the swapping loop (*SL*). According to our measurements, Δ*L*_2_ = (66.9–31.0) = 35.9 nm. Because the increment in contour length associated with the unfolding of Ntd is Δ*L* = 29.3 nm (Fig. 2*D*), it follows that the swapping loop extension is 6.6 nm (35.9–29.3 nm), involving 16 amino acids. Bearing all this in mind, a close inspection of the Ntd structure reveals that only the loop connecting the β2-β3 strands is composed of 15 amino acids (all other loops are significantly shorter), and is placed right at the N terminus of the crystallin Ntd. This suggests that β1-β2 strands, encompassing amino acids Ile-3 to Cys-18, form a structural motif that is a very likely candidate to swap (Fig. 5, *A* and *B*).

According to this hypothesis, we constructed a topological model for the domain-swapped protein construct that is consistent with our findings (Fig. 5*C*). This model clearly identifies the swapping motifs between two Ntds and highlights the sequence of structural rearrangements the polyprotein construct undergoes when exposed to mechanical force. Crucially, the model provides a visual representation of the points of application of force during each unfolding step. Remarkably, the mechanical stability of Ntd2 of the swapped structure (∼35 pN) is significantly lower than that corresponding to the unfolding of the Ntd in the regular trajectories. According to our topological model (Fig. 5*C*), such a drastic change in mechanical resistance can be explained by a change in pulling direction. It is indeed well reported that the direction of force propagation has a strong effect on the mechanical stability of proteins (42–44). To prove the hypothesis, we constructed a circular permutant that mimics the pulling direction of Ntd2 in the context of the swapped trajectories. Mechanical unfolding of this circular permutant of Ntd2 (data not shown) confirmed the low mechanical stability of Ntd2 when the pulling direction was changed.

## Discussion

Using our polyprotein approach, we covalently linked two HγD-crys monomers, each of which was a multidomain protein with high sequence identity. Such strategy results in a high effective local protein concentration, *a priori* making it much more vulnerable to unfolding and aggregation (27, 45). Domain swapping has been identified as a general possible initial mechanism for protein misfolding (41, 46). Lacking the crystal structure, direct identification of these low-probability conformations remains challenging using traditional biophysical techniques. By contrast, single-molecule techniques are ideally suited to detect rare events (22, 26). In this vein, pioneering experiments using AFM revealed domain swapping-based misfolding events within individual polyproteins made out of eight identical repeating domains of the well characterized I27th module of the giant protein titin, (I27)_8_ (23). These results were further supported by single molecule FRET experiments, suggesting that sequence diversity might be an evolutionary strategy to avoid misfolding in multidomain proteins (27, 47).

Our single-molecule experiments readily identified a previously elusive domain-swapped HγD-crys dimer conformation. On the basis of the independent unfolding of both domains in the multidomain protein, both exhibiting distinct mechanical stabilities, and according to their unfolding sequence within the swapped form, we suggest that the swapped element most likely corresponds to the β1 and β2 strands of the N terminus domain. At this point, we cannot rule out the possibility that the swapping motif encompasses the terminal β7 strand instead. In this case, the swapping loop linking the β7 strand with β6 would also be composed of 15 amino acids. However, the fact that the loop has secondary structure (α helix), together with the observation that the β1 and β2 strands exhibit the higher B-factor (suggesting the lowest stability, PDB code 1HK0) within the Ntd, strongly favors the hypothesis of β1 and β2 strands, encompassing amino acids Ile-3 to Cys-18, being, in fact, the swapping motif.

**FIGURE 6.**
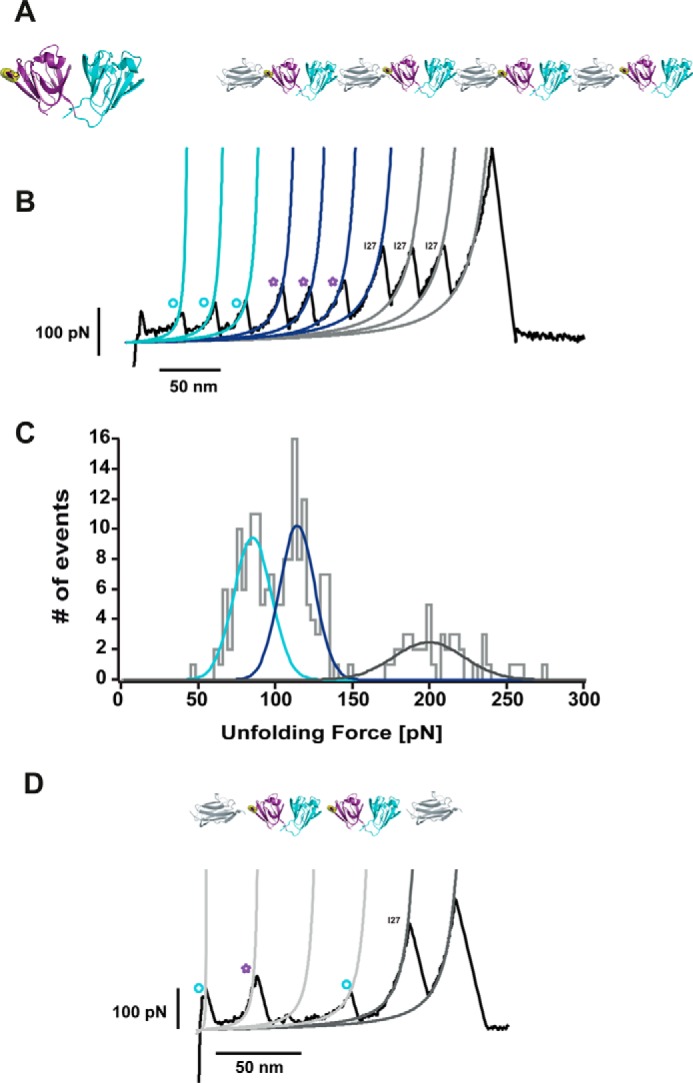
**The R14C mutation exhibits misfolding trajectories akin to those corresponding to the wild-type HγD-crys.** *A*, the mechanical stability of the (I27-HγD-crys_R14C_)_4_ polyprotein exhibits the same unfolding pattern (*B*) as the wild-type (I27-HγD-crys)_4_ polyprotein. *C*, the Ctd unfolds first at 83.7 ± 12.2 pN (*blue fits*, *circles*), followed by the unfolding of the Ntd at 112.6 ± 7.28 pN (*purple fits*, *stars*). Finally, the I27 modules unfold at higher forces, 197.9 ± 22.9 pN (*gray fits*). *D*, to investigate whether the R14C mutation triggered misfolding mechanisms that differ from that observed for the wild-type protein, we engineered the [I27-(HγD-crys_R14C_)_2_-I27] polyprotein. Akin to the wild-type case, the misfolding trajectories were reminiscent of a domain swap scenario. In these single-molecule experiments, we did not identify any other alternative misfolding mechanism, probably induced by the putative presence of a disulfide bond.

These results are in contrast with molecular dynamics simulations that identify a domain-swapped structure that occurs within the Ctd instead (20). For the swap to occur, the Ntd of the protein has first to unfold, at least partially (Fig. 5*A*). In this vein, it is intriguing that most of the congenital cataractogenic mutations in the γ-crystallins (T4P, L5S/F9S, R14C, P23S/P23T, W42R, V75D, and R76R), which readily trigger the protein to unfold, occur in the N terminus (10, 48–51); incidentally, a great number of these occur within the β1 and β2 strands. In particular, the R14C mutation has been described to trigger juvenile-onset cataract, in which aggregation is thought to occur through disulfide bonds between adjacent domains (50). To investigate whether such a distinct aggregation mechanism could be identified at the single-molecule level, we constructed the (I27-HγD-crys_R14C_)_4_ and [I27-(HγD-crys_R14C_)_2_-I27] polyproteins, the latter with two HγD-crys_R14C_ flanking monomers. Pulling on these proteins (Fig. 6) provided similar results as the wild-type protein. Crucially, although anomalous trajectories consistent with a domain-swapped conformation were also captured with similar probability, no further misfolding mechanisms compatible with a putative disulfide bond, which would significantly shorten the protein extensibility, were observed. Qualitatively, our results point toward the same direction as the biochemical experiments conducted in the bulk that postulate the presence of a swapped conformation with a folded Ctd and a partially (un)folded Ntd. It is interesting to point out that, for the swap to occur, the mechanically resistant Ntd needs to partially unfold. Bulk experiments have indeed demonstrated that the Ntd has a lower thermodynamic stability than the Ctd counterpart. However, the stability of these domains is context-specific (33), and slight variations in the relative stability of both domains, often through particular mutations, might ultimately determine the probability for the domain swap to occur.

**FIGURE 7.**
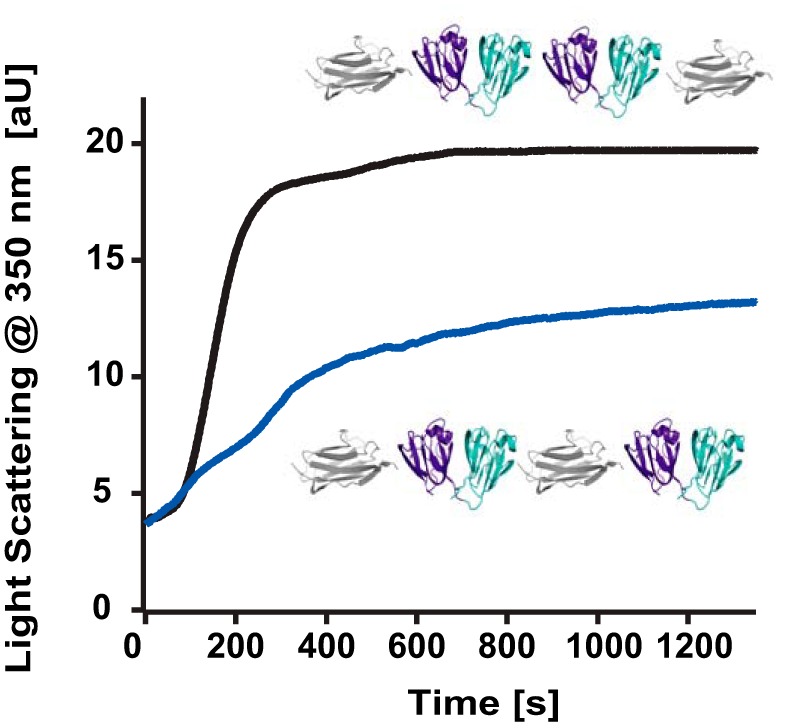
**Kinetics of light scattering at 350 nm.** The [I27-(HγD-crys)_2_-I27] polyprotein (*black*) aggregates more (and faster) than the related (HγD-crys-I27)_2_ polyprotein.

More generically, domain swapping has been identified as a general possible initial mechanism for protein misfolding (41, 46), often being the conformational seed for the aggregation of a variety of proteins, including the human prion protein (52), β2-microglobulin (53, 54), cystatin C (55), amyotrophic lateral sclerosis (1), and antitrypsin deficiency (2). Therefore, it is tempting to speculate that the domain-swapped conformations we identified at the single-molecule level could exhibit a higher propensity to aggregate. To test this hypothesis, we conducted aggregation bulk assays in which we measured the light scattering of the [I27-(HγD-crys)_2_-I27] polyprotein as a function of time and compared it with the value corresponding to the homologous [I27-(HγD-crys)]_2_ polyprotein, where each HγD-crys is separated by an I27 module (Fig. 7). These results show a higher tendency to aggregate than the constructs composed of two HγD-crys neighboring domains.

It is premature to conclude that the previously elusive domain-swapped conformation we identify is the unique cause for the aggregation. However, the analysis of human cataracts reveals the presence of insoluble species that are not structured precipitates but, rather, resemble the aggregates and polymers of partially folded intermediates associated with inclusion bodies or other forms of protein misfolding (5, 56). Therefore, it is at least enticing to hypothesize that the swapped dimer we captured at the single-molecule level might represent the onset seed of a particular (perhaps one of many) polymerization pathway leading to high molecular weight species associated with cataracts. Lacking structural characterization, probably because of the fast aggregation kinetics and the dynamic nature of the system, our experiments provide a structural insight into the first stages of misfolding that evaded capture using ensemble techniques. Together, our results provide a new piece in the puzzle of understanding the early mechanisms underlying protein misfolding occurring at the single-molecule level.

## Author Contributions

S. G. M. and J. M. F. designed the research. S. G. M. and A. E. M. B. performed the AFM experiments. C. B. and A. L. conducted molecular biology. D. G. contributed to the structural model. J. P. C. conducted the aggregation experiments. S. G. M. analyzed the data and wrote the paper. All authors contributed to revising and editing the manuscript.

## Footnotes

2 The abbreviations used are:

HγD-crys: human γ D crystallin
Ctd: C-terminal domain
Ntd: N-terminal domain
AFM: atomic force microscopy.

## References

[1] Mulligan V. K., and Chakrabartty A. (2013) Protein misfolding in the late-onset neurodegenerative diseases: common themes and the unique case of amyotrophic lateral sclerosis. Proteins 81, 1285–1303 23508986 10.1002/prot.24285

[2] Carrell R. W., and Lomas D. A. (2002) α1-antitrypsin deficiency: a model for conformational diseases. N. Engl. J. Med. 346, 45–53 11778003 10.1056/NEJMra010772

[3] Yamasaki M., Sendall T. J., Pearce M. C., Whisstock J. C., and Huntington J. A. (2011) Molecular basis of α1-antitrypsin deficiency revealed by the structure of a domain-swapped trimer. EMBO Rep. 12, 1011–1017 21909074 10.1038/embor.2011.171 PMC3185345

[4] Liu Y., and Eisenberg D. (2002) 3D domain swapping: as domains continue to swap. Protein Sci. 11, 1285–1299 12021428 10.1110/ps.0201402 PMC2373619

[5] Wang Y., and King J. (2010) in Protein Misfolding Diseases: Current and Emerging Principles and Therapies (Ramirez-Alvarado M., Kelly J. W., and Dobson C. M., eds.), pp. 487–515, Wiley, Hoboken, New Jersey

[6] Slingsby C., Wistow G. J., and Clark A. R. (2013) Evolution of crystallins for a role in the vertebrate eye lens. Protein Sci. 22, 367–380 23389822 10.1002/pro.2229 PMC3610043

[7] Slingsby C., and Wistow G. J. (2014) Functions of crystallins in and out of lens: roles in elongated and post-mitotic cells. Prog. Biophys. Mol. Biol. 115, 52–67 24582830 10.1016/j.pbiomolbio.2014.02.006 PMC4104235

[8] Moreau K. L., and King J. A. (2012) Protein misfolding and aggregation in cataract disease and prospects for prevention. Trends Mol. Med. 18, 273–282 22520268 10.1016/j.molmed.2012.03.005 PMC3621977

[9] Slingsby C., and Clout N. J. (1999) Structure of the crystallins. Eye 13, 395–402 10627816 10.1038/eye.1999.113

[10] Serebryany E., and King J. A. (2014) The βγ-crystallins: native state stability and pathways to aggregation. Prog. Biophys. Mol. Biol. 115, 32–41 24835736 10.1016/j.pbiomolbio.2014.05.002 PMC4438767

[11] Hejtmancik J. F. (2008) Congenital cataracts and their molecular genetics. Semin. Cell Dev. Biol. 19, 134–149 18035564 10.1016/j.semcdb.2007.10.003 PMC2288487

[12] Graw J. (2009) Genetics of crystallins: cataract and beyond. Exp. Eye. Res. 88, 173–189 19007775 10.1016/j.exer.2008.10.011

[13] Basak A., Bateman O., Slingsby C., Pande A., Asherie N., Ogun O., Benedek G. B., and Pande J. (2003) High-resolution X-ray crystal structures of human γD crystallin (1.25 A) and the R58H mutant (1.15 A) associated with aculeiform cataract. J. Mol. Biol. 328, 1137–1147 12729747 10.1016/s0022-2836(03)00375-9

[14] Flaugh S. L., Kosinski-Collins M. S., and King J. (2005) Contributions of hydrophobic domain interface interactions to the folding and stability of human γD-crystallin. Protein Sci. 14, 569–581 15722442 10.1110/ps.041111405 PMC2279286

[15] Kosinski-Collins M. S., and King J. (2003) *In vitro* unfolding, refolding, and polymerization of human γ D crystallin, a protein involved in cataract formation. Protein Sci. 12, 480–490 12592018 10.1110/ps.0225503 PMC2312441

[16] Kosinski-Collins M. S., Flaugh S. L., and King J. (2004) Probing folding and fluorescence quenching in human γ D crystallin Greek key domains using triple tryptophan mutant proteins. Protein Sci. 13, 2223–2235 15273315 10.1110/ps.04627004 PMC2279819

[17] Papanikolopoulou K., Mills-Henry I., Thol S. L., Wang Y., Gross A. A., Kirschner D. A., Decatur S. M., and King J. (2008) Formation of amyloid fibrils *in vitro* by human γD-crystallin and its isolated domains. Mol. Vis. 14, 81–89 18253099 PMC2267726

[18] Sandilands A., Hutcheson A. M., Long H. A., Prescott A. R., Vrensen G., Löster J., Klopp N., Lutz R. B., Graw J., Masaki S., Dobson C. M., MacPhee C. E., and Quinlan R. A. (2002) Altered aggregation properties of mutant γ-crystallins cause inherited cataract. EMBO J. 21, 6005–6014 12426373 10.1093/emboj/cdf609 PMC137201

[19] Mohr B. G., Dobson C. M., Garman S. C., and Muthukumar M. (2013) Electrostatic origin of *in vitro* aggregation of human γ-crystallin. J. Chem. Phys. 139, 121914 24089726 10.1063/1.4816367 PMC3745490

[20] Das P., King J. A., and Zhou R. (2011) Aggregation of γ-crystallins associated with human cataracts via domain swapping at the C-terminal β-strands. Proc. Natl. Acad. Sci. U.S.A. 108, 10514–10519 21670251 10.1073/pnas.1019152108 PMC3127930

[21] Fisher T. E., Oberhauser A. F., Carrion-Vazquez M., Marszalek P. E., and Fernandez J. M. (1999) The study of protein mechanics with the atomic force microscope. Trends Biochem. Sci. 24, 379–384 10500301 10.1016/s0968-0004(99)01453-x

[22] Fernandez J. M., Garcia-Manyes S., and Dougan L. (2010) Force-clamp spectroscopy of single proteins. Springer Ser. Chem. Ph. 96, 317–335

[23] Oberhauser A. F., Marszalek P. E., Carrion-Vazquez M., and Fernandez J. M. (1999) Single protein misfolding events captured by atomic force microscopy. Nat. Struct. Biol. 6, 1025–1028 10542093 10.1038/14907

[24] Dougan L., Li J., Badilla C. L., Berne B. J., and Fernandez J. M. (2009) Single homopolypeptide chains collapse into mechanically rigid conformations. Proc. Natl. Acad. Sci. U.S.A. 106, 12605–12610 19549822 10.1073/pnas.0900678106 PMC2722357

[25] Yu H., Liu X., Neupane K., Gupta A. N., Brigley A. M., Solanki A., Sosova I., and Woodside M. T. (2012) Direct observation of multiple misfolding pathways in a single prion protein molecule. Proc. Natl. Acad. Sci. U.S.A. 109, 5283–5288 22421432 10.1073/pnas.1107736109 PMC3325692

[26] Borgia A., Williams P. M., and Clarke J. (2008) Single-molecule studies of protein folding. Annu. Rev. Biochem. 77, 101–125 18412537 10.1146/annurev.biochem.77.060706.093102

[27] Borgia M. B., Borgia A., Best R. B., Steward A., Nettels D., Wunderlich B., Schuler B., and Clarke J. (2011) Single-molecule fluorescence reveals sequence-specific misfolding in multidomain proteins. Nature 474, 662–665 21623368 10.1038/nature10099 PMC3160465

[28] Carrion-Vazquez M., Oberhauser A. F., Fowler S. B., Marszalek P. E., Broedel S. E., Clarke J., and Fernandez J. M. (1999) Mechanical and chemical unfolding of a single protein: a comparison. Proc. Natl. Acad. Sci. U.S.A. 96, 3694–3699 10097099 10.1073/pnas.96.7.3694 PMC22356

[29] Schlierf M., Li H., and Fernandez J. M. (2004) The unfolding kinetics of ubiquitin captured with single-molecule force-clamp techniques. Proc. Natl. Acad. Sci. U.S.A. 101, 7299–7304 15123816 10.1073/pnas.0400033101 PMC409913

[30] Popa I., Kosuri P., Alegre-Cebollada J., Garcia-Manyes S., and Fernandez J. M. (2013) Force dependency of biochemical reactions measured by single-molecule force-clamp spectroscopy. Nat. Protoc. 8, 1261–1276 23744288 10.1038/nprot.2013.056 PMC4676941

[31] Ramanujam V., Kotamarthi H. C., and Ainavarapu S. R. K. (2014) Ca^2+^ binding enhanced mechanical stability of an archaeal crystallin. PloS ONE 9, e94513 24728085 10.1371/journal.pone.0094513 PMC3984160

[32] Mayr E. M., Jaenicke R., and Glockshuber R. (1994) Domain interactions and connecting peptides in lens crystallins. J. Mol. Biol. 235, 84–88 8289268 10.1016/s0022-2836(05)80017-8

[33] Mayr E. M., Jaenicke R., and Glockshuber R. (1997) The domains in γB-crystallin: identical fold-different stabilities. J. Mol. Biol. 269, 260–269 9191069 10.1006/jmbi.1997.1033

[34] Flaugh S. L., Kosinski-Collins M. S., and King J. (2005) Interdomain side-chain interactions in human γD crystallin influencing folding and stability. Protein Sci. 14, 2030–2043 16046626 10.1110/ps.051460505 PMC2279314

[35] Sikora M., and Cieplak M. (2011) Mechanical stability of multidomain proteins and novel mechanical clamps. Proteins 79, 1786–1799 21465555 10.1002/prot.23001

[36] Peng Q., and Li H. (2009) Domain insertion effectively regulates the mechanical unfolding hierarchy of elastomeric proteins: toward engineering multifunctional elastomeric proteins. J. Am. Chem. Soc. 131, 14050–14056 19746906 10.1021/ja903589t

[37] Fernandez V. I., Kosuri P., Parot V., and Fernandez J. M. (2009) Extended Kalman filter estimates the contour length of a protein in single molecule atomic force microscopy experiments. Rev. Sci. Instrum. 80, 113104 19947714 10.1063/1.3252982

[38] Bax B., Lapatto R., Nalini V., Driessen H., Lindley P. F., Mahadevan D., Blundell T. L., and Slingsby C. (1990) X-ray analysis of β B2-crystallin and evolution of oligomeric lens proteins. Nature 347, 776–780 2234050 10.1038/347776a0

[39] Itoh K., and Sasai M. (2008) Cooperativity, connectivity, and folding pathways of multidomain proteins. Proc. Natl. Acad. Sci. U.S.A. 105, 13865–13870 18772375 10.1073/pnas.0804512105 PMC2544545

[40] Li H., and Fernandez J. M. (2003) Mechanical design of the first proximal Ig domain of human cardiac titin revealed by single molecule force spectroscopy. J. Mol. Biol. 334, 75–86 14596801 10.1016/j.jmb.2003.09.036

[41] Bennett M. J., Schlunegger M. P., and Eisenberg D. (1995) 3D domain swapping: a mechanism for oligomer assembly. Protein Sci. 4, 2455–2468 8580836 10.1002/pro.5560041202 PMC2143041

[42] Carrion-Vazquez M., Li H., Lu H., Marszalek P. E., Oberhauser A. F., and Fernandez J. M. (2003) The mechanical stability of ubiquitin is linkage dependent. Nat. Struct. Biol. 10, 738–743 12923571 10.1038/nsb965

[43] Dietz H., Berkemeier F., Bertz M., and Rief M. (2006) Anisotropic deformation response of single protein molecules. Proc. Natl. Acad. Sci. U.S.A. 103, 12724–12728 16908850 10.1073/pnas.0602995103 PMC1568916

[44] Brockwell D. J., Paci E., Zinober R. C., Beddard G. S., Olmsted P. D., Smith D. A., Perham R. N., and Radford S. E. (2003) Pulling geometry defines the mechanical resistance of a β-sheet protein. Nat. Struct. Biol. 10, 731–737 12923573 10.1038/nsb968

[45] Han J. H., Batey S., Nickson A. A., Teichmann S. A., and Clarke J. (2007) The folding and evolution of multidomain proteins. Nat. Rev. Mol. Cell Biol. 8, 319–330 17356578 10.1038/nrm2144

[46] Rousseau F., Schymkowitz J. W., and Itzhaki L. S. (2003) The unfolding story of three-dimensional domain swapping. Structure 11, 243–251 12623012 10.1016/s0969-2126(03)00029-7

[47] Wright C. F., Teichmann S. A., Clarke J., and Dobson C. M. (2005) The importance of sequence diversity in the aggregation and evolution of proteins. Nature 438, 878–881 16341018 10.1038/nature04195

[48] Jung J., Byeon I. J., Wang Y., King J., and Gronenborn A. M. (2009) The Structure of the cataract-causing P23T mutant of human γ D-crystallin exhibits distinctive local conformational and dynamic changes. Biochemistry 48, 2597–2609 19216553 10.1021/bi802292q PMC2722838

[49] Pande A., Annunziata O., Asherie N., Ogun O., Benedek G. B., and Pande J. (2005) Decrease in protein solubility and cataract formation caused by the Pro23 to Thr mutation in human γ D-crystallin. Biochemistry 44, 2491–2500 15709761 10.1021/bi0479611

[50] Pande A., Pande J., Asherie N., Lomakin A., Ogun O., King J. A., Lubsen N. H., Walton D., and Benedek G. B. (2000) Molecular basis of a progressive juvenile-onset hereditary cataract. Proc. Natl. Acad. Sci. U.S.A. 97, 1993–1998 10688888 10.1073/pnas.040554397 PMC15742

[51] Santhiya S. T., Shyam Manohar M., Rawlley D., Vijayalakshmi P., Namperumalsamy P., Gopinath P. M., Löster J., and Graw J. (2002) Novel mutations in the γ-crystallin genes cause autosomal dominant congenital cataracts. J. Med. Genet. 39, 352–358 12011157 10.1136/jmg.39.5.352 PMC1735119

[52] Knaus K. J., Morillas M., Swietnicki W., Malone M., Surewicz W. K., and Yee V. C. (2001) Crystal structure of the human prion protein reveals a mechanism for oligomerization. Nat. Struct. Biol. 8, 770–774 11524679 10.1038/nsb0901-770

[53] Eakin C. M., Attenello F. J., Morgan C. J., and Miranker A. D. (2004) Oligomeric assembly of native-like precursors precedes amyloid formation by β-2 microglobulin. Biochemistry 43, 7808–7815 15196023 10.1021/bi049792q

[54] Liu C., Sawaya M. R., and Eisenberg D. (2011) β2-Microglobulin forms three-dimensional domain-swapped amyloid fibrils with disulfide linkages. Nat. Struct. Mol. Biol. 18, 49–55 21131979 10.1038/nsmb.1948 PMC3058263

[55] Wahlbom M., Wang X., Lindström V., Carlemalm E., Jaskolski M., and Grubb A. (2007) Fibrillogenic oligomers of human cystatin C are formed by propagated domain swapping. J. Biol. Chem. 282, 18318–18326 17470433 10.1074/jbc.M611368200

[56] Wetzel R. (1994) Mutations and off-pathway aggregation of proteins. Trends Biotechnol. 12, 193–198 7764903 10.1016/0167-7799(94)90082-5

